# Perspective Toward Machine Learning Implementation in Pediatric Medicine: Mixed Methods Study

**DOI:** 10.2196/40039

**Published:** 2022-11-17

**Authors:** Natasha Alexander, Catherine Aftandilian, Lin Lawrence Guo, Erin Plenert, Jose Posada, Jason Fries, Scott Fleming, Alistair Johnson, Nigam Shah, Lillian Sung

**Affiliations:** 1 Division of Haematology/Oncology The Hospital for Sick Children Toronto, ON Canada; 2 Division of Hematology/Oncology Department of Pediatrics Stanford University Palo Alto, CA United States; 3 Program in Child Health Evaluative Sciences Peter Gilgan Centre for Research and Learning Toronto, ON Canada; 4 Department of Systems Engineering and Computing Universidad del Norte Barranquilla Colombia; 5 Stanford Center for Biomedical Informatics Research Department of Biomedical Data Science Stanford University Palo Alto, CA United States

**Keywords:** machine learning, clinical utilization, preferences, qualitative interviews

## Abstract

**Background:**

Given the costs of machine learning implementation, a systematic approach to prioritizing which models to implement into clinical practice may be valuable.

**Objective:**

The primary objective was to determine the health care attributes respondents at 2 pediatric institutions rate as important when prioritizing machine learning model implementation. The secondary objective was to describe their perspectives on implementation using a qualitative approach.

**Methods:**

In this mixed methods study, we distributed a survey to health system leaders, physicians, and data scientists at 2 pediatric institutions. We asked respondents to rank the following 5 attributes in terms of implementation usefulness: the clinical problem was common, the clinical problem caused substantial morbidity and mortality, risk stratification led to different actions that could reasonably improve patient outcomes, reducing physician workload, and saving money. Important attributes were those ranked as first or second most important. Individual qualitative interviews were conducted with a subsample of respondents.

**Results:**

Among 613 eligible respondents, 275 (44.9%) responded. Qualitative interviews were conducted with 17 respondents. The most common important attributes were risk stratification leading to different actions (205/275, 74.5%) and clinical problem causing substantial morbidity or mortality (177/275, 64.4%). The attributes considered least important were reducing physician workload and saving money. Qualitative interviews consistently prioritized implementations that improved patient outcomes.

**Conclusions:**

Respondents prioritized machine learning model implementation where risk stratification would lead to different actions and clinical problems that caused substantial morbidity and mortality. Implementations that improved patient outcomes were prioritized. These results can help provide a framework for machine learning model implementation.

## Introduction

Machine learning has had growing popularity in clinical settings related to the widespread adoption of electronic health records [1-3], combined with increasing data storage and computational ability [4]. In this setting, machine learning can be useful for multiple purposes including (1) to facilitate diagnoses, as in pathology [5,6] and radiology [7]; (2) to make predictions about outcomes for risk stratification; and (3) to improve resource utilization by anticipating volumes of patients or services [8]. However, despite the initial enthusiasm around machine learning in health care, domain experts have expressed caution [9,10]. Similar information technology solutions have commonly failed to be implemented or provide utility [11].

An important consideration impacting utility is choosing the clinical setting and problem in which a machine learning model is to be implemented [11]. A machine learning model’s predictions need to augment current approaches in a way that is meaningful and actionable without introducing excessive burden. It is important to carefully plan a machine learning model’s implementation because the costs of model deployment are considerable. Such costs may include resources required to develop and maintain the machine learning model, training of the intended model users regarding how to access and interpret the model’s predictions, and support to help users implement the results into practice [12,13].

Given these costs, a systematic approach for determining which machine learning models should be prioritized for implementation into clinical practice may be valuable. In determining priorities, it would be important to involve key stakeholders at the institution in which deployment is planned. We chose to survey 2 pediatric centers, 1 in the United States with a more established biomedical informatics program, and 1 in Canada with a less established biomedical informatics program, to gain insight into whether experience and expertise affected preferences for machine learning model prioritization. Consequently, the primary objective was to determine the health care attributes respondents at 2 pediatric institutions rate as important when prioritizing machine learning model implementation. The secondary objective was to describe their perspectives on machine learning model implementation using a qualitative approach.

## Methods

### Study Design and Setting

This was a mixed methods study that included a quantitative and a qualitative component. The institutions were The Hospital for Sick Children (SickKids) in Toronto, Ontario, Canada, and Lucile Packard Children’s Hospital in Palo Alto, California, United States.

### Participants

We included health system leaders, physicians, and data scientists at SickKids and Lucile Packard Children’s Hospital at the time of survey distribution. We excluded trainees.

### Procedures

The survey was developed by the study team based on their impression of health care attributes respondents might consider to be important; the machine learning–focused questions are presented as Multimedia Appendix 1. Potential participants were identified through organizational emailing lists. The quantitative survey was distributed by email and participants completed the survey in REDCap [14]. The survey asked respondents to indicate whether they were health system leaders, physicians, or data scientists; respondents could indicate multiple categories. Demographic variables included clinical specialty (if applicable), years employed following completion of training, and gender.

We then asked about their knowledge of artificial intelligence on a 5-point Likert scale ranging from 1 (no knowledge at all) to 5 (a lot of knowledge). We asked them to rate their understanding of how machine learning models are built and interpreted, and how statistics are conducted and interpreted, using 5-point Likert scales ranging from 1 (no understanding) to 5 (fully understand). We asked if they had decision-making ability to implement artificial intelligence initiatives within their work environment, and how many machine learning models had been deployed at their institutions in the last 5 years.

The next section asked respondents to rank the following 5 clinical problem and implementation consequence attributes in terms of whether machine learning implementation would be useful: “the clinical problem being solved is common,” “the clinical problem causes substantial morbidity or mortality,” “risk stratification would lead to different clinical actions that could reasonably improve patient outcomes,” “implementing the model could reduce physician workload,” and “implementing the model could save money.” Important attributes were defined as those ranked as most important or second most important (rank of 1 or 2) by respondents. The survey then asked 2 open-ended questions focused on clinical areas where being able to accurately predict an outcome might be useful, and clinical areas in which prioritization or reorganization of waitlists might be useful. Finally, the survey asked whether they would be willing to participate in a qualitative interview.

For the qualitative aspect, we purposively sampled respondents to maximize variation by institution and self-rated understanding of machine learning. Semistructured interviews were conducted using Zoom (Zoom Video Communications, Inc.) or Microsoft Teams by a member of the SickKids team (EP) with expertise in the conduct of qualitative interviews. Respondents were asked to list 3 scenarios in which a machine learning model for risk stratification could be useful and then to state which scenario was the most important to implement first and the rationale for the choice. They were then asked how they would feel about using a machine learning model for risk stratification as opposed to their current approach, and to describe concerns they had about using a machine learning model to guide patient care. The interviews were recorded and transcribed verbatim.

### Analysis

The data from the quantitative survey from SickKids and Lucile Packard Children’s Hospital were compared using the Fisher exact test. Analyses were performed in R (R Core Team) using RStudio version 3.6.1 [15,16].

The analysis of qualitative data was performed according to the principles of grounded theory methodology; data collection and analysis occurred concurrently. Qualitative transcripts were analyzed by 2 independent reviewers (NA and EP) using the constant comparative method to develop a theoretical framework for respondents’ perspectives of machine learning that are grounded in their individual experiences and understandings. Sampling was continued until saturation was reached, which was defined as the point in which no new themes emerged from the data.

### Ethics Approval

The study was approved by the Research Ethics Board at SickKids. The need for Institutional Review Board approval was waived by Lucile Packard Children’s Hospital as the data collection was performed by SickKids personnel. For the quantitative survey, completion of the survey was considered implied consent to study participation. For the qualitative component, respondents provided verbal consent to participate.

## Results

**Figure 1:**
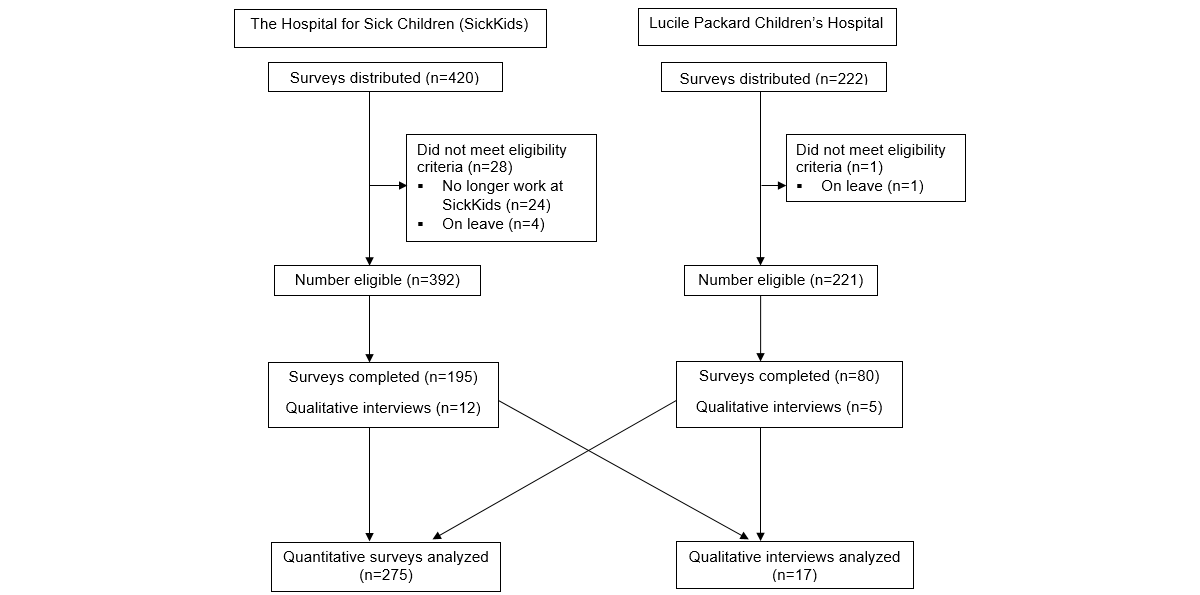
CONSORT (Consolidated Standards of Reporting Trials) diagram of participant identification, selection, and participation.

The quantitative survey was distributed at SickKids between November 1, 2021, and January 6, 2022 and at Lucile Packard Children’s Hospital between March 15, 2022, and April 12, 2022. Among 613 eligible respondents, 275 (44.9%) responded. Figure 1 shows the participant identification and selection flowchart, including the number participating in the qualitative interviews when saturation was reached.

Table 1 presents the demographic characteristics of respondents; physician specialty (*P*<.001) and years from completion of training (*P*=.006) were significantly different between the 2 institutions. The majority of respondents were physicians (165/195, 84.6%, at SickKids and 73/80, 91.3%, at Lucile Packard Children’s Hospital). The number of respondents who had decision-making ability to implement artificial intelligence initiatives was 99/195 (50.8%) at SickKids and 41/80 (51.3%) at Lucile Packard Children’s Hospital. Most respondents did not know the number of machine learning models deployed at their institution over the last 5 years (137/195, 70.3%, at SickKids and 53/80, 66.3%, at Lucile Packard Children’s Hospital).

Table 2 illustrates respondents’ self-perceived knowledge of artificial intelligence and understanding of machine learning and statistics. There were no statistically significant differences in these ratings by institution (artificial intelligence knowledge, *P*=.93; machine learning development and interpretation, *P*=.72; statistics conduct and interpretation, *P*=.19). The percentage of respondents who stated they had “moderate” or “a lot” of artificial intelligence knowledge was 17.9% (35/195) at SickKids and 17.5% (14/80) at Lucile Packard Children’s Hospital. Multimedia Appendix 2 compares respondent characteristics by those who self-rated their artificial intelligence knowledge as high (score of 4 or 5 on the 5-point Likert scale) versus not high across institutions. Those who self-rated their knowledge as high were significantly more likely to be males (*P*=.02) and nonphysicians (*P*=.006). The percentage of respondents who stated they understood machine learning development and interpretation at a “moderate” level or “fully” was 15.9% (31/195) at SickKids and 11.3% (9/80) at Lucile Packard Children’s Hospital. Across both institutions, the percentage who stated their understanding of machine learning was “none” or “very little” was 146/275 (53.1%). Conversely, the percentage of respondents who stated they understood statistics conduct and interpretation at a “moderate” level or “fully” was 54.4% (106/195) at SickKids and 42.5% (34/80) at Lucile Packard Children’s Hospital. Across both institutions, the percentage who stated their understanding of statistics was “none” or “very little” was 30/275 (10.9%).

**Table 1:** Demographic characteristics of participants at 2 pediatric institutions (N=275).

Characteristic			SickKids (n=195), n (%)		Lucile Packard Children’s Hospital (n=80), n (%)		*P* value
Male gender			93 (47.7)		35 (43.8)		.64
**Professional role^a^**							
	Physician	165 (84.6)		73 (91.3)		.20	
	Health system leader	22 (11.3)		17 (21.3)		.05	
	Data scientist	15 (7.7)		2 (2.5)		.18	
**Physician specialty**							<.001
	Hematology oncology	33 (16.9)		14 (17.5)			
	General medicine	21 (10.8)		7 (8.8)			
	Critical care medicine	11 (5.6)		12 (15.0)			
	Emergency medicine	14 (7.2)		0 (0)			
	Cardiology	9 (4.6)		7 (8.8)			
	Neurology	11 (5.6)		3 (3.8)			
	Endocrinology and metabolism	10 (5.1)		6 (7.5)			
	Gastroenterology	9 (4.6)		0 (0)			
	Respirology	4 (2.1)		4 (5.0)			
	Infectious disease	2 (1.0)		5 (6.3)			
	Surgery	0 (0)		6 (7.5)			
	Adolescent medicine	6 (3.1)		0 (0)			
	Other	20 (10.3)		7 (8.8)			
	Not known	45 (23.1)		9 (11.3)			
**Years from completion of training**							.006
	<1	6 (3.1)		0 (0)			
	1-4	38 (19.5)		5 (6.3)			
	5-10	38 (19.5)		25 (31.3)			
	11+	113 (57.9)		50 (62.5)			
Decision-making ability to implement artificial intelligence initiatives			99 (50.8)		41 (51.3)		>.99
**Number of machine learning models deployed at institution in last 5 years**							.43
	None	31 (15.9)		11 (13.8)			
	1	7 (3.6)		6 (7.5)			
	2-4	14 (7.2)		9 (11.3)			
	5-10	2 (1.0)		1 (1.3)			
	11+	4 (2.1)		0 (0)			
	Do not know	137 (70.3)		53 (66.3)			

^a^Respondent may choose more than 1 option and thus, numbers do not add to 100%.

**Table 2:** Self-rating of knowledge of artificial intelligence and understanding of machine learning and statistics.

Areas		SickKids (n=195), n (%)	Lucile Packard Children’s Hospital (n=80), n (%)	*P*-value
**Artificial intelligence knowledge**				.93
	None	10 (5.1)	5 (6.3)	
	Very little	67 (34.4)	30 (37.5)	
	Some	83 (42.6)	31 (38.8)	
	Moderate	30 (15.4)	11 (13.8)	
	A lot	5 (2.6)	3 (3.8)	
**Machine learning development and interpretation**				.72
	None	44 (22.6)	18 (22.5)	
	Very little	56 (28.7)	28 (35.0)	
	Somewhat	64 (32.8)	25 (31.3)	
	Moderate	24 (12.3)	8 (10.0)	
	Fully	7 (3.6)	1 (1.3)	
**Statistics conduct and interpretation**				.19
	None	4 (2.1)	1 (1.3)	
	Very little	18 (9.2)	7 (8.8)	
	Somewhat	67 (34.4)	38 (47.5)	
	Moderate	78 (40.0)	29 (36.3)	
	Fully	28 (14.4)	5 (6.3)	

Table 3 reveals the proportion of respondents who ranked each attribute as important (ranked first or second among the 5 attributes) for prioritization of machine learning models. There were no significant differences in these proportions by institution for any of the 5 attributes (Table 3). Across both sites, the most common important attributes were risk stratification leading to different actions (205/275, 74.5%) and clinical problem causes substantial morbidity or mortality (177/275, 64.4%). The attributes considered least important were “implementing the model could reduce physician workload” (40/275, 14.5%) and “implementing the model could save money” (13/275, 4.7%). The median importance scores for both institutions combined are also shown in Table 3 (where lower is more important).

**Table 3:** Ranked as important^a^ by respondents for prioritization of machine learning.

Attributes considered important	SickKids (n=195), n (%)	Lucile Packard Children’s Hospital (n=80), n (%)	*P*-value	Median importance score (IQR)^b^
The clinical problem being solved is common	66 (33.8)	35 (43.8)	.16	3 (2-3)
The clinical problem causes substantial morbidity or mortality	133 (68.2)	44 (55.0)	.05	2 (2-3)
Risk stratification would lead to different clinical actions that could reasonably improve patient outcomes	145 (74.4)	60 (75.0)	>.99	1 (1-2)
Implementing the model could reduce physician workload	29 (14.9)	11 (13.8)	.96	4 (3-4)
Implementing the model could save money	11 (5.6)	2 (2.5)	.42	5 (4-5)

^a^Important defined as attributes ranked as most important or second most important (rank of 1 or 2) in terms of whether a machine learning model would be useful.

^b^Across both institutions.

Table 4 shows the themes and subthemes from the qualitative interviews. Perceived benefits of machine learning model implementation included facilitating decision making in complex scenarios, supporting less experienced clinicians, reducing cognitive load, and reducing cognitive bias. It was also expressed that machine learning models can potentially improve the quality of care through standardization, more effective triage, and facilitating precision medicine. Finally, machine learning models had the potential to reduce physician workload. However, perceived challenges of machine learning model implementation included the potential for algorithmic bias, lack of transparency and trust, and failure to incorporate clinical expertise. Machine learning model implementation might also adversely affect quality of care and respondents spoke about the need to evaluate the impact of machine learning model implementation. Practical concerns raised about machine learning model implementation included challenges incorporating the model into the clinical workflow and questions about accountability in the event of poor outcomes arising from machine learning model–directed actions. Finally, uncertainty about the physician’s role was identified. When asked to prioritize 1 clinical scenario for machine learning model implementation, the rationale for choosing which scenario to implement consistently related to impact on patient outcomes: “most benefit to kids,” “leading cause of death,” and “implications can be extremely serious.”

Multimedia Appendix 3 illustrates examples of clinical areas that could be prioritized for machine learning initiatives identified from the quantitative survey.

**Table 4:** Perspectives of machine learning implementation in pediatric medicine from qualitative interviews.

Themes and subthemes					Example quotations
Benefits of machine learning implementation					
	**Facilitates decision making**				
		Complex scenario	*To me was very disturbing scenario where a very complex child with a number of issues, [...] Having some kind of system which alerts physicians who are directly involved as to not any in their own domains, but in other domains’ risk would be helpful*		
		Support less experienced clinicians	*Well, you know where I see potential strength is not so much for the highly experienced physician, but more for the person who’s starting out [...] and just doesn't have that experience base yet.*		
		Reduce cognitive load	*It can offload some of the cognitive load. So yeah, absolutely. I mean there's many times you find yourself in the middle of the night very tired, half groggy and trying to make a decision and kind of going back and forth in your brain. You know, for like half an hour - should I do this or that?*		
		Reduce cognitive bias	*[...]* * it's not that it replaces your judgment, it supplements another sense.* *...* *your decisions informed no matter by your experience but it's informed by thousands of experiences, computed even more times to see all the possibilities and then come up with a best sort of path forward. The most likely scenario. And understanding that it is not a perfect prediction but it's a much more* *...* *It's where that big data come in, right? It's really powered by real knowledge. It's not personal perceptions or personal experience, which is very biased and skewed.*		
	**Improve quality of care**				
		Standardize care	*There probably is some significant interpersonal variability in terms of interpreting the guidelines and then decision making around management, and so if we could use machine learning so that there’s less of that, all the while providing I guess more accurate or better care. I think that would be very helpful.*		
		More effective triage	*I feel like if we were able to use machine learning to risk stratify so that kids who are at higher risk could get more timely access to a referral. Recognizing that in this particular situation, certainly early diagnosis and management can really impact the trajectory of a child’s outcome. I think that would be helpful.*		
		Facilitate precision medicine	*And what I mean by that if you look at it, look at a population of babies who were all born, say at 25 weeks. There will be individual differences that should [...] be detectable by machine learning or artificial intelligence. So instead of treating every baby as simply a member of the population, I can sort of drill down onto specific physiological and clinical factors for that baby, [...] get closer to the idea of personalized medicine.*		
	**Reduce physician workload**				
		Freeing up time for physicians	*If it was really useful, then maybe it would free me up to do things that only I can do.*		
**Challenges with** **machine** **learning implementation**					
	**Hinders decision making**				
		Algorithmic bias	*It's all about the biases like built into the system and how it's learned the data that you're putting in, and then how you get that out and how it would either pick up on our own biases, or like pre-existing, whether those are like systemic like sort of racial, ethnic or gendered biases [...] And so then that's not really helping us.*		
		Lack of transparency and trust	*Understanding what it is doing: like if it's doing things that I can't follow or don't understand, I'm going to be less to trust its opinion [...] I want to understand how it came to that decision so I can ask myself if I agree.*		
		Not incorporating clinical expertise into decisions	*I think it's like all the tools we have in medicine that if you use it appropriately, it can be incredibly powerful. But if it's used as a, you know, let me abandon all my other skills and I'll just follow this kind of direction, it potentially could be harmful, so I think a lot of thought will be needed.* *I mean in some ways it helps to predict, but I think I've always been a little skeptical about machine learning because biology and people do not follow an algorithm, they don’t follow a formula.*		
	**Negative impact on quality of care**				
		Need for outcome evaluation	*[...] looking at what the outcomes are and that we're actually improving patient care. So if we're admitting more but the outcomes are the same and the return visits are the same, then did it really matter and are we improving patient care or we just increasing cost to the system? And so, I think it needs constant evaluation, just like anything else that we do...*		
		Data quality	*Of course, you know your outcome or the recommendation, or how machine learning is used is always only as good as the input, right?*		
	**Practical concerns**				
		Challenges in workflow implementation	*I guess there’s going to be some learning curve. How do we use it? Is it feasible? Is it on my iPhone? Do I have to go into certain area, how fast will it take me to get the response and along with the interface, how friendly is the interface? You know things that are related to stuff that we have not seen yet.*		
		Accountability	*The challenge with machine learning over clinical decision rules is right now with the accountability piece and it's just getting to what that's going to be like. We don't blame, you know, the lab test or the lab. You know, if we don't pick it up. But right now, I think people feeling if they go against it, what does that mean and do we have to add like admit everybody or treat everybody based on that, knowing that like you alluded on the first question that it is a probability [...] So what does that mean for the provider thing choose to ignore it versus if they choose to follow it in harm happens*		
	**Physician role**				
		Uncertainty in physician role	*On the other hand, you know, maybe it also kind of takes away a little bit from like, I guess there's a fear of what exactly is the doctor's role. If the computer can do a better job at diagnosing then I can*		

## Discussion

In this mixed methods study, we found that the attributes most commonly listed as important for machine learning model implementation were risk stratification leading to different actions that could reasonably improve patient outcomes and a clinical problem that causes substantial morbidity or mortality. Few respondents considered reducing physician workload and saving money as important. We also found that important attributes were similar at the 2 institutions despite different levels of biomedical informatic program establishment and different health care systems.

The wide range of recommended areas for machine learning model implementation highlights the need for prioritization given the likely limited capacity to develop, deploy, and monitor machine learning models, even at large institutions with mature bioinformatics programs. This study is important as it provides a framework by which institutional leaders could make decisions about which machine learning models to prioritize for implementation. While we found that risk stratification that improves patient outcomes was the most common important attribute, additional considerations include actions that would arise from high- and low-risk labels, evidence that differential actions will improve outcomes, and identifying ideal thresholds for risk categorization. Even once a model is deployed, ongoing monitoring of model performance and the impact of model deployment on patient care and clinical workflows are additional postimplementation considerations.

While we evaluated attribute importance across respondent types, Wears and Berg [11] previously discussed the complex relationship between decision makers, beneficiaries of a machine learning solution, and those who shoulder the burden of implementation. They noted that a mismatch between these individuals can lead to failure. More specifically, it is often the administrator who is the decision maker and recipient of benefits, while it is the clinician who often shoulders the burden of implementation [11]. Anticipation and acknowledgement of conflicting perspectives will be required during the prioritization process among stakeholder types.

We also found that across both institutions, respondents had greater confidence in their understanding of statistics and relatively lower confidence in their understanding of machine learning. These perspectives did not differ between the 2 institutions despite different levels of establishment of their biomedical informatic programs. Our results suggest that across pediatric medicine in general, more education focused on machine learning is required during training and continuing education.

Our results complement the work of others who have highlighted the requirements of clinical decision support including those based on machine learning. Items important to consider include the need to avoid black boxes, excessive time requirement, and complexity in addition to ensuring relevance, respect, and scientific validity [17-19]. It also accompanies work demonstrating that barriers to adoption of artificial intelligence are not restricted to clinicians but also include parents [20,21]. It may also be useful to compare our findings with studies conducted outside of pediatric medicine. We found that the main anticipated benefits of machine learning implementation were facilitation of decision making, improvement in quality of care, and reduction in physician workload. Compared with our findings, benefits and challenges associated with artificial intelligence were similar in ophthalmology, dermatology, radiology, optometry, and surgery [22,23]. However, our study is unique because of the consideration of how to prioritize problems for implementation, a pragmatic consideration in developing a clinical program. In addition, the focus on pediatrics may be important as the nature of clinical problems, perspectives, and stakeholders can differ between pediatric and adult patient populations.

The strengths of this study include its mixed methods design and inclusion of 2 different pediatric institutions by country and establishment of their biomedical informatic programs. However, our results should be interpreted in light of their limitations. We had a relatively low response rate; respondents were likely biased in favor of interest in machine learning. Thus, nonrespondents likely would have had lower familiarity with machine learning and likely would have had less strong opinions about attributes considered important for machine learning prioritization. We also had a greater proportion of physicians than system leaders or data scientists; these groups may have different priorities or implementation concerns.

In conclusion, respondents prioritized machine learning model implementation where risk stratification would lead to different actions and clinical problems that caused substantial morbidity and mortality. Implementations that improved patient outcomes were prioritized. These results can help provide a framework for prioritizing machine learning model implementation.

## Appendix

Multimedia Appendix 1 Quantitative survey administered.

Multimedia Appendix 2 Comparison of participants with artificial intelligence knowledge high versus not high (N=275).

Multimedia Appendix 3 Examples of recommendations of areas in pediatric care that should be prioritized for machine learning from quantitative survey.
