# Supplementary material for: Perspective Toward Machine Learning Implementation in Pediatric Medicine: Mixed Methods Study
Source: JMIR Med Inform. 2022 Nov 17;10(11):e40039. doi: 10.2196/40039 (PMC9716421; doi:10.2196/40039)
Supplement: Multimedia Appendix 1 [file medinform_v10i11e40039_app1.docx]

**Appendix 1: Quantitative Survey Administered^a^**

| 1. In trying to determine the settings in which a machine learning model would be clinically useful to predict the risk of an outcome, please rank the following according to their importance relative to one another, where 1 = most important and 5 = least important. Assume that the model will be reliable, acceptable and stable over time in our population. | | | | | |
| --- | --- | --- | --- | --- | --- |
|  | 1  Most important | 2 | 3 | 4 | 5  Least important |
| 1. The clinical problem being solved (for examples, sepsis) is common |  |  |  |  |  |
| 1. The clinical problem causes substantial morbidity or mortality |  |  |  |  |  |
| 1. Risk stratification would lead to different clinical actions that could reasonably improve patient outcomes |  |  |  |  |  |
| 1. Implementing the model could reduce physician workload |  |  |  |  |  |
| 1. Implementing the model could save money |  |  |  |  |  |
| 1. What clinical areas do you think being able to accurately predict an outcome might be useful? Please be as descriptive as possible. | | | | | |
| 1. What clinical areas do you think being able to prioritize or reorganize queues/waitlists might be useful? Please be as descriptive as possible. | | | | | |

^a^ Represents a portion of quantitative survey focused on machine learning prioritization
