# Supplementary material for: Perspective Toward Machine Learning Implementation in Pediatric Medicine: Mixed Methods Study
Source: JMIR Med Inform. 2022 Nov 17;10(11):e40039. doi: 10.2196/40039 (PMC9716421; doi:10.2196/40039)
Supplement: Multimedia Appendix 2 [file medinform_v10i11e40039_app2.docx]

**Appendix 2: Comparison of Participants with Artificial Intelligence Knowledge High vs. Not High^a^ (N=275)**

| Characteristic | Artificial Intelligence Knowledge High  (n=49) | Artificial Intelligence Knowledge Not High  (n=226) | *P* Value |
| --- | --- | --- | --- |
| Male Gender | 31 (63.3%) | 97 (42.9%) | .02 |
| Physician | 36 (73.5%) | 202 (89.4%) | .006 |
| Years from Completion of Training |  |  | .74 |
| <1 year | 2 (4.1%) | 4 (1.8%) |  |
| 1-4 years | 8 (16.3%) | 35 (15.5%) |  |
| 5-10 years | 12 (24.5%) | 51 (22.6%) |  |
| 11+ years | 27 (55.1%) | 136 (60.2%) |  |

**^a^** High dichotomized as score of 4 or 5 as rated on a 5-point Likert scale ranging from 1 = “no understanding” to 5 = “fully understand”
