# Supplementary material for: Perspective Toward Machine Learning Implementation in Pediatric Medicine: Mixed Methods Study
Source: JMIR Med Inform. 2022 Nov 17;10(11):e40039. doi: 10.2196/40039 (PMC9716421; doi:10.2196/40039)
Supplement: Multimedia Appendix 3 [file medinform_v10i11e40039_app3.docx]

**Appendix 3: Examples of Recommendations of Areas in Pediatric Care that should be Prioritized for Machine Learning from Quantitative Survey**

|  | **Outpatient Clinic** | **Emergency Room** | **Inpatient Wards** | **Critical Care** |
| --- | --- | --- | --- | --- |
| **Clinical features** | Headache  Lymphadenopathy  Failure to thrive | Abdominal pain  Fever  Injury  Mental health crisis | Shock  Seizure  Jaundice | Cardiac arrest  Respiratory distress  Trauma |
| **Investigations** | Hematopathology  EEG  Sleep studies | X-ray interpretation  Diagnostic test choice | Pathology  Genetic testing  Novel non-invasive tools | MRI or CT  Echocardiogram  Biomarkers and vital signs |
| **Diagnosis** | Diabetes  IBD  Food allergy | Sepsis  Fractures  Child abuse | Infection  Sickle cell disease  Leukemia | NEC  Stroke  Burns |
| **Treatment** | Radiotherapy  Non-compliance Clinical response to medication | Asthma treatment  Fracture management  Sepsis management | Pain management  Stem cell transplant  Drug dosing and duration | Dialysis timing  Sepsis management  Tumor lysis syndrome |
| **Prognosis** | Need for admission  Late morbidity in cancer patients  Outcome after stroke | Need for ICU care  Need for admission  Likelihood of deterioration | Post-operative complications  Length of stay  Readmission risk | Risk of re-intubation  Response to diuretics  Effectiveness of ventilation |
| **Workflow** | Triage virtual or in-person care  Outpatient referrals  Surgery waiting list | Triage patients  Need for resources and staff  Need for admission | Discharge planning  Ward round scheduling  Verbal ordering and charting EMR | Operating room lists  Transfers between hospitals  Physician burden |

^a^ Abbreviations: EEG – electroencephalogram; ICU - intensive care unit; NEC - necrotizing enterocolitis; HIE - hypoxic ischemic encephalopathy; MRI - magnetic resonance imaging; CT - computerized tomography; EMR - electronic medical records
